# Supplementary material for: From sequence to enzyme mechanism using multi-label machine learning
Source: BMC Bioinformatics. 2014 May 19;15:150. doi: 10.1186/1471-2105-15-150 (PMC4229970; doi:10.1186/1471-2105-15-150)
Supplement: Additional file 2 — Java code of ml2db. Additional file ml2db_code.tar.gz contains the Java source code to run the multi-label machine learning experiments and save the results to database. The code’s Javadoc is included. [file 1471-2105-15-150-S2.zip › additional file 2/ml2db/ml2db/doc/test/ml2db/mulan/learn/CrossEvaluatorTest.html]

CrossEvaluatorTest


JavaScript is disabled on your browser.


- Overview
- Package
- Class
- Use
- Tree
- Deprecated
- Index
- Help

- Prev Class
- Next Class

- Frames
- No Frames

- All Classes

- Summary:
- Nested |
- Field |
- Constr |
- Method

- Detail:
- Field |
- Constr |
- Method


test.ml2db.mulan.learn

## Class CrossEvaluatorTest

- java.lang.Object
- - junit.framework.Assert
  - - junit.framework.TestCase
    - - test.ml2db.mulan.learn.CrossEvaluatorTest

- All Implemented Interfaces:
  :   junit.framework.Test

  ---

    

  ```
  public class CrossEvaluatorTest
  extends junit.framework.TestCase
  ```

  Class

  Version:
  :   8 Jun 2010

  Author:
  :   Luna De Ferrari ldf(at)st-andrews.ac.uk

- - ### Constructor Summary

    Constructors

    Constructor and Description

    `CrossEvaluatorTest()`
  - ### Method Summary

    Methods

    | Modifier and Type | Method and Description |
    | --- | --- |
    | `static CrossEvaluator` | `getMulanEvaluator2()` |
    | `static CrossEvaluator` | `getMulanEvaluatorTestSet()` |
    | `void` | `setUp()` |
    | `void` | `testCrossValidate1()` |
    | `void` | `testCrossValidate2()` |
    | `void` | `testRunningTime()` public void testEvaluationResults() { MulanEvaluator evaluator = MulanEvaluatorTest.getMulanEvaluator(); assertFalse(evaluator == null); if (evaluator != null) { Evaluation[] results = evaluator.getEvaluationResults(); assertEquals(10, results.length); Evaluation e0 = results[0]; System.out.println(e0.toString()); java.util.List measures = e0.getMeasures(); assertEquals(22, measures.size()); // hamming loss Measure m0 = measures.get(0); assertEquals("Hamming Loss", m0.getName()); double value = m0.getValue(); System.out.println("MulanEvaluatorTest. |

    - ### Methods inherited from class junit.framework.TestCase

      `countTestCases, getName, run, run, runBare, setName, toString`
    - ### Methods inherited from class junit.framework.Assert

      `assertEquals, assertEquals, assertEquals, assertEquals, assertEquals, assertEquals, assertEquals, assertEquals, assertEquals, assertEquals, assertEquals, assertEquals, assertEquals, assertEquals, assertEquals, assertEquals, assertEquals, assertEquals, assertEquals, assertEquals, assertFalse, assertFalse, assertNotNull, assertNotNull, assertNotSame, assertNotSame, assertNull, assertNull, assertSame, assertSame, assertTrue, assertTrue, fail, fail, failNotEquals, failNotSame, failSame, format`
    - ### Methods inherited from class java.lang.Object

      `equals, getClass, hashCode, notify, notifyAll, wait, wait, wait`

- - ### Constructor Detail


    - #### CrossEvaluatorTest

      ```
      public CrossEvaluatorTest()
      ```
  - ### Method Detail


    - #### setUp

      ```
      public void setUp()
                 throws java.lang.Exception
      ```

      **Overrides:**
      :   `setUp` in class `junit.framework.TestCase`

      Throws:
      :   `java.lang.Exception`


    - #### testCrossValidate1

      ```
      public void testCrossValidate1()
      ```


    - #### testCrossValidate2

      ```
      public void testCrossValidate2()
      ```


    - #### testRunningTime

      ```
      public void testRunningTime()
      ```

      public void testEvaluationResults() { MulanEvaluator evaluator =
      MulanEvaluatorTest.getMulanEvaluator(); assertFalse(evaluator == null);
      if (evaluator != null) { Evaluation[] results =
      evaluator.getEvaluationResults(); assertEquals(10, results.length);
      Evaluation e0 = results[0]; System.out.println(e0.toString());
      java.util.List measures = e0.getMeasures(); assertEquals(22,
      measures.size());
      // hamming loss Measure m0 = measures.get(0);
      assertEquals("Hamming Loss", m0.getName()); double value = m0.getValue();
      System.out.println("MulanEvaluatorTest. Hamming loss value: " + value);
      // assertTrue((Double.parseDouble("0.1") > value) && (value < //
      Double.parseDouble("0.2"))); assertEquals(0.0, m0.getIdealValue()); }
      }


    - #### getMulanEvaluator2

      ```
      public static CrossEvaluator getMulanEvaluator2()
      ```


    - #### getMulanEvaluatorTestSet

      ```
      public static CrossEvaluator getMulanEvaluatorTestSet()
      ```


- Overview
- Package
- Class
- Use
- Tree
- Deprecated
- Index
- Help

- Prev Class
- Next Class

- Frames
- No Frames

- All Classes

- Summary:
- Nested |
- Field |
- Constr |
- Method

- Detail:
- Field |
- Constr |
- Method
